# Retinoic Acid Treatment Mitigates PM2.5-Induced Type 2 Inflammation: Insights into Modulation of Innate Immune Responses

**DOI:** 10.3390/ijms25073856

**Published:** 2024-03-29

**Authors:** Hyun-Joo Lee, Dong-Kyu Kim

**Affiliations:** 1 Institute of New Frontier Research, Hallym University College of Medicine, Chuncheon 24252, Republic of Korea; leekul79@gmail.com; 2 Department of Otorhinolaryngology-Head and Neck Surgery, Chuncheon Sacred Heart Hospital, Hallym University College of Medicine, Chuncheon 24253, Republic of Korea

**Keywords:** innate lymphoid cells, nasal polyp, particulate matter, retinoic acid, rhinosinusitis

## Abstract

Some studies have demonstrated the effects of particulate matter (PM) on chronic rhinosinusitis with nasal polyps (CRSwNP) development, as well as the therapeutic role of retinoic acid (RA) in nasal polypogenesis. However, the immunologic effect of PM in innate lymphoid cells (ILCs) and the exact mechanism of the therapeutic effect of RA remain unclear. Therefore, the present study investigated the effects of fine-dust-induced inflammation in CRSwNP and the mechanisms of the therapeutic effect of RA. PM2.5 exposure exacerbated pathological damage in the nasal mucosa of mice with nasal polyps (NP) via upregulation of type 2 inflammation. Additionally, PM2.5 exposure increased the expression of type 2 cytokines and epithelial-cell-derived cytokines (IL-33 and IL-25) significantly, as well as the ILC populations in human-NP-derived epithelial cells (HNECs). Moreover, RA supplementation significantly increased the expression of ILCreg in Lin−CD45+CD127+ cells, which in turn increased the levels of the anti-inflammatory cytokine IL-10. The findings suggest that PM2.5 exposures could aggravate the CRSwNP type 2 inflammation, and RA treatment may ameliorate fine-dust-induced inflammation by modulating the innate immune response.

## 1. Introduction

Chronic rhinosinusitis (CRS) develops when acute sinusitis is not properly treated or when there are repeated episodes of acute inflammation, defined as localized inflammation of the upper airway and paranasal sinuses that lasts for at least 12 weeks [1]. Epidemiological studies have shown that CRS occurs in approximately 4–11% of the general population and is associated with a significant decline in quality of life [2,3,4,5]. Currently, there are two phenotypes, based on the presence of nasal polyps (NP) on nasal endoscopy: CRS with nasal polyps (CRSwNP) and CRS without nasal polyps (CRSsNP). CRSwNP is characterized by a type 2 immune response, whereas CRSsNP is characterized by a mixed immune response [6,7,8]. Generally, patients with CRSwNP show more severe symptoms and poorer prognoses than those with CRSsNP [9,10,11].

The expeditious progression of technology, coupled with accelerated urbanization and population growth, has brought air pollution to the forefront of academic discourse, particularly in light of its profound implications for public health. Additionally, studies suggest that being exposed to secondhand smoke increases the likelihood of contracting respiratory infections. The substances in cigarette smoke have the potential to hinder the immune system’s effectiveness, though the specific mechanisms behind this interference are not yet fully understood. A weakened immune response resulting from such interference might contribute to the recurrence of respiratory infections. One recent study determined that in children experiencing frequent respiratory infections, passive smoke exposure impairs the production of interferon-gamma in their adenoids [12].

Some of the main outdoor and indoor air pollutants are fine dust and volatile organic compounds [13]. Fine dust refers to fine particles barely visible to the naked eye, and is an air pollutant that enters the body through the respiratory tract and causes harmful damage to various organs. Fine dust is classified as PM10 (<10 μm) or PM2.5 (<2.5 μm), based on diameter. The nose is the most important passageway for air, and it plays an important role in filtering and humidifying air. Generally, the nose filters particles larger than 10 μm in diameter via the synergistic activity of mucus and cilia in the nasal mucosa. However, particles smaller than 2.5 μm can escape the nasal mucosa and travel down the lower airways. Although the pathogenesis of CRS is unclear, some studies have suggested that environmental factors are associated with its development [14,15,16]. For example, PM10 exposure significantly increased the IL-33/ST2 pathway-mediated type 2 immune response in patients with CRSwNP [17]. Moreover, fine dust particles can be absorbed into the bloodstream and cause several health problems [18,19]. According to the World Health Organization European Air Quality Guidelines published in 1987, fine dust is classified as a carcinogen [20].

The group 2 innate lymphoid cell (ILC2) is known as a type of innate cell found among innate lymphoid cells and lacking the expression of makers identified as T cells, B cells, dendritic cells, granulocytes, and macrophages. They are lineage-negative cells capable of producing type 2 cytokines, including IL-4, IL-5, and IL-13, while expressing the surface markers CD45 and CD127, as well as the transcription factor GATA3. It is well-known that, in allergic airway inflammatory diseases such as asthma and CRSwNP, ILC2s play important roles in the induction and exacerbation of inflammation [21]. The ILC2 also initiates and amplifies airway inflammation by activating various cells, including eosinophils, B cells, mast cells, macrophages, fibroblasts, and epithelial cells.

Recently, some studies also suggested that retinoic acid (RA) could exert beneficial effects on CRSwNP. One study showed that RA suppressed type 2 inflammation by altering ILC2 secretion of IL-10 in NP tissues, thereby promoting regulatory ILCs [22]. Another study demonstrated that RA increases fibrinolysis in CRSwNP and may regulate nasal polypogenesis [23]. Given the regulatory effects of RA on the type 2 immune response, it is crucial to investigate the therapeutic effects and molecular mechanisms of RA in CRSwNP. Accordingly, we hypothesized that RA may exert therapeutic effects on the fine-dust-induced exacerbation of acute CRSwNP. Therefore, the aim of the present study was to investigate the effect of fine-dust exposure on the ILC2-related immunologic status of patients with CRSwNP. In addition, we investigated the mechanisms of the therapeutic effects of RA in fine-dust-exacerbated ILC2-related inflammation.

## 2. Results

### 2.1. Effects of Fine Dust in Mouse Model of Nasal Polyp

**Figure 1:**
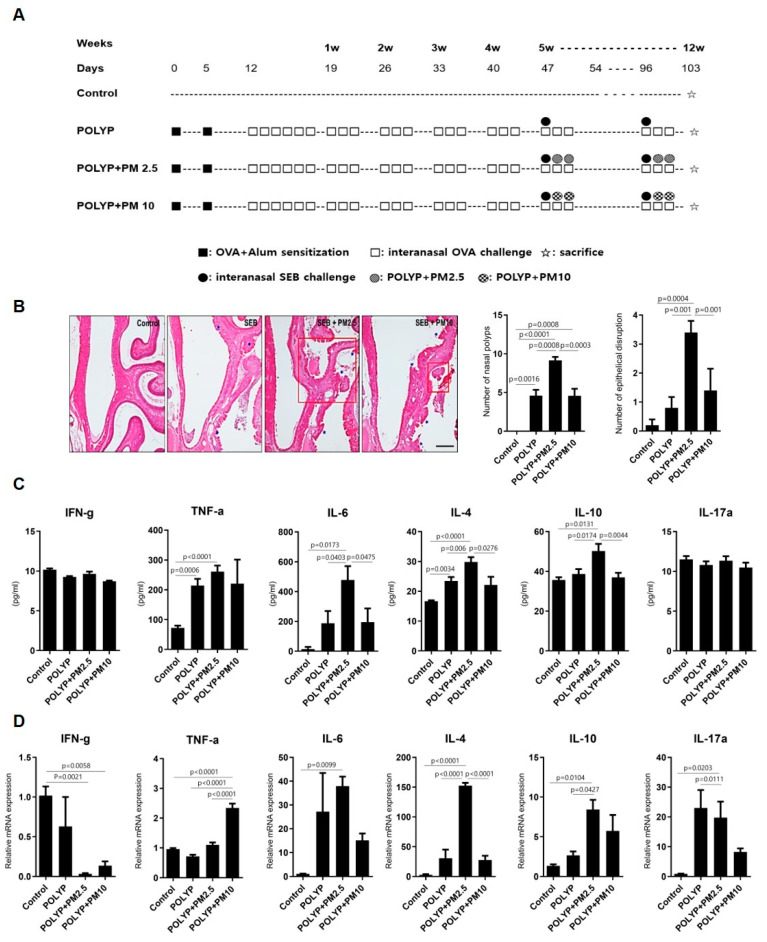
PM2.5 and PM10 exposure exacerbates nasal polypogenesis in a mouse model of nasal polyposis (NP). (**A**) Schematic diagram of the experimental protocol of murine NP model. (**B**) Representative hematoxylin–eosin-stained images of sinonasal spaces and polyps from indicated groups (scale bar 100 mm). Blue stars denote the NP lesions. Nasal polyps and epithelial disruptions were counted and averaged in five different HPFs. POLYP = nasal polyp model; POLYP + PM2.5 = PM2.5-exposed NP model; POL-YP + PM10 = PM10-exposed NP model. (**C**) IL-4, IL-10, IL-6 IFN-γ, TNF-α, and IL-17a expression in nasal lavage fluid were analyzed using CBA assay. (**D**) IL-4, IL-10, IL-6 IFN-γ, TNF-α, and IL-17a mRNA expression in nasal tissue was analyzed using qRT-PCR. All results are compared among groups (n = 5 for control, n = 5 for NP, n = 7 for NP + PM2.5, and n = 7 for NP + PM10) and presented as means ± standard error of mean (SEM). Statistical significance was determined using one-way ANOVA tests.

To evaluate the effect of fine dust (PM2.5 and PM10) on CRSwNP, a mouse model of nasal polyposis was established (Figure 1A). Exposure of mice with NP to fine dust (PM2.5) significantly exacerbated nasal polypogenesis and disruption of epithelial cells compared with that found in the unexposed NP model group. However, PM10 exposure did not significantly exacerbate nasal polypogenesis in mice with NP, compared with results in the unexposed NP model group (Figure 1B). Additionally, PM2.5 exposures significantly increased interleukin-6 (IL-6), IL-4, and IL-10 protein levels in the nasal fluid of mice with NP, compared with those in the unexposed NP model group (Figure 1C) (*p* = 0.0403, *p* = 0.006, *p* = 0.0174). Moreover, PM2.5 exposure significantly upregulated IL-4 mRNA levels (*p* < 0.0001) and downregulated the mRNA expression of interferon gamma (IFN-γ), compared with that in the unexposed NP model group (Figure 1D). Although PM10 stimulation significantly upregulated TNF-α mRNA expression, no significant differences were noted in the expression of other inflammatory factors between the PM10-exposed NP group and the unexposed NP model group (Figure 1C,D). Collectively, these results suggest that PM2.5 exposure exacerbates pathological damage in mice with NP and activates inflammatory cytokines.

### 2.2. Effects of PM2.5 Exposure on the Expression of Inflammatory Cytokines and Epithelial-Cell-Derived Cytokines in Human Nasal Epithelial Cells

**Figure 2:**
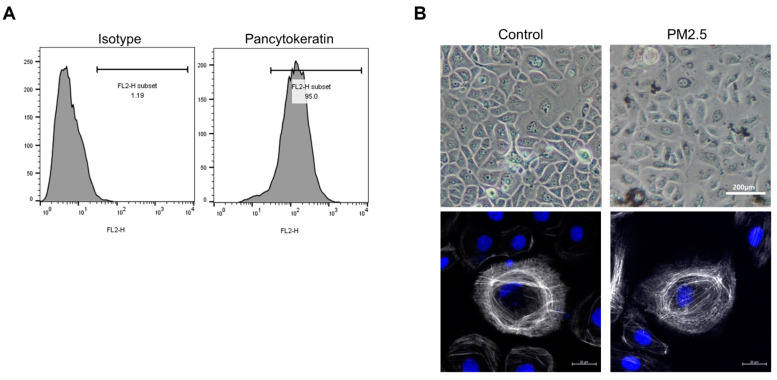

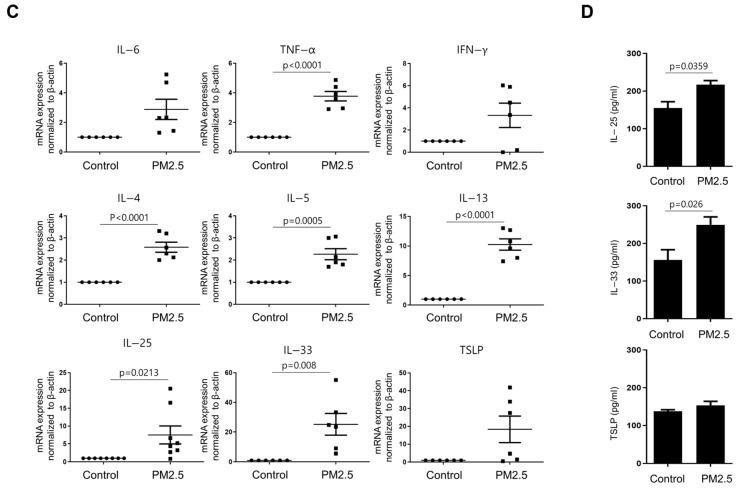
Expression of signature inflammatory cytokines and epithelial-cell-derived cytokines in human nasal epithelial cells (HNECs) after PM2.5 exposures. (**A**) We used pan-cytokeratin AE1/AE3 Ab to confirm the deposit of PM2.5 in cultured HNECs from nasal tissue. To confirm this, we compared the staining results of the isotype control antibody as the positive signal. (**B**) Representative phase contrast images of stained HNECs. Scale bars = 200 μm (upper panels). Immunofluorescence analysis of PM2.5-treated HNECs. The boxed region indicates internalized PM2.5. Scale bars = 20 μm (lower panels). (**C**) The mRNA expression levels of IL-4, IL-5, IL-6, IL-13, IL-25, IL-33, TNF-α, IFN-γ, and TSLP in PM2.5-treated HNECs were analyzed using qRT-PCR. (**D**) The protein expression levels of IL-25, IL-33, and TSLP were analyzed in PM2.5-exposed HNECs. The graphic data represent the means ± standard error of mean (SEM; n = 5). Statistical significance was determined using the Mann–Whitney U test.

To validate the effects of fine dust (PM2.5) on nasal polypogenesis in humans, human nasal epithelial cells (HNECs) were isolated from patients with CRSwNP and exposed to PM2.5 (50 μg/mL) for 24 h (Figure 2A). HNECs were stained with phalloidin (red) and DAPI (blue) to investigate the cellular localization of PM2.5, and we found that PM2.5 was localized in the cellular structure (Figure 2B). Additionally, we examined the effect of PM2.5 exposures on the severity of CRSwNP. PM2.5 stimulations significantly increased the mRNA expression of TNF-α, IL-4, IL-5, and IL-13 in HNECs (Figure 2C) (*p* < 0.0001, *p* = 0.0005, *p* < 0.0001). Additionally, PM2.5 exposures significantly increased the mRNA expression of major epithelial-cell-derived cytokines, such as IL-25 and IL-33, in HNECs (Figure 2D) (*p* = 0.0213, *p* = 0.008). Overall, these results indicate that PM2.5 exposure increases the expression of signature cytokines related to type 2 inflammation in patients with CRSwNP.

### 2.3. Effect of Exposure of Human Nasal Epithelial Cells to PM2.5 on the Immunological Activity of Innate Lymphoid Cells

**Figure 3:**
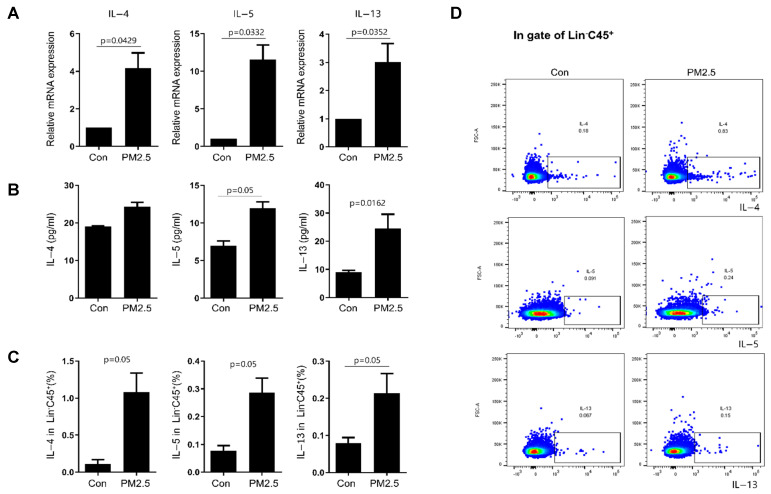
PM2.5 conditioned media (PM2.5-CM) obtained from human nasal epithelial cells after PM2.5 stimulations on innate lymphoid cells (ILCs). Lin cells were cultured in PM2.5-CM for 3 days. (**A**) The mRNA expression of IL-4, IL-5, and IL-13 in Lin cells was analyzed using qRT-PCR. (**B**) The protein expression of IL-4, IL-5, and IL-13 in culture supernatants was determined using CBA assay. (**C**,**D**) The percentage of IL-4, IL-5, and IL-13 in Lin cells was determined using flow cytometry. The graphic data represent the means ± standard error of mean (SEM; n = 3). Con = control. Statistical significance was determined using the Mann–Whitney U test.

Given that PM2.5 stimulation induced the production of epithelial-cell-derived cytokines and type 2 immune responses in CRSwNP, we examined whether PM2.5 stimulations can affect the type 2 immunological activity of innate lymphoid cells (ILCs). To explore the effect of PM2.5, human lineage (Lin) cells derived from tonsillar mononuclear cells (TMCs) were stimulated with PM2.5-conditioned media (PM2.5-CM). PM2.5-CM was obtained following treatment of HNECs with PM2.5 (50 μg/mL) at 37 °C for 24 h. The expression of IL-4, IL-5, and IL-13 in Lin cells was analyzed. Stimulation with PM2.5-CM significantly increased the mRNA expression of IL-4, IL-5, and IL-13 and the protein expression of IL-5 and IL-13 in Lin cells, compared with that in the control media (Con-CM)-stimulated group (Figure 3A,B). Moreover, PM2.5-CM stimulation significantly increased the expression of IL-4, IL-5, and IL-13 in Lin cells; all of these data are shown with a *p*-value of 0.05 (Figure 3C,D). Collectively, these results indicate that HNEC stimulation by PM2.5 may promote type 2 immune response via enhanced cytokine expression.

### 2.4. Effects of Exposure of Human Nasal Epithelial Cells to PM2.5 on the Induction of Type 2 Innate Lymphoid Cells

**Figure 4:**
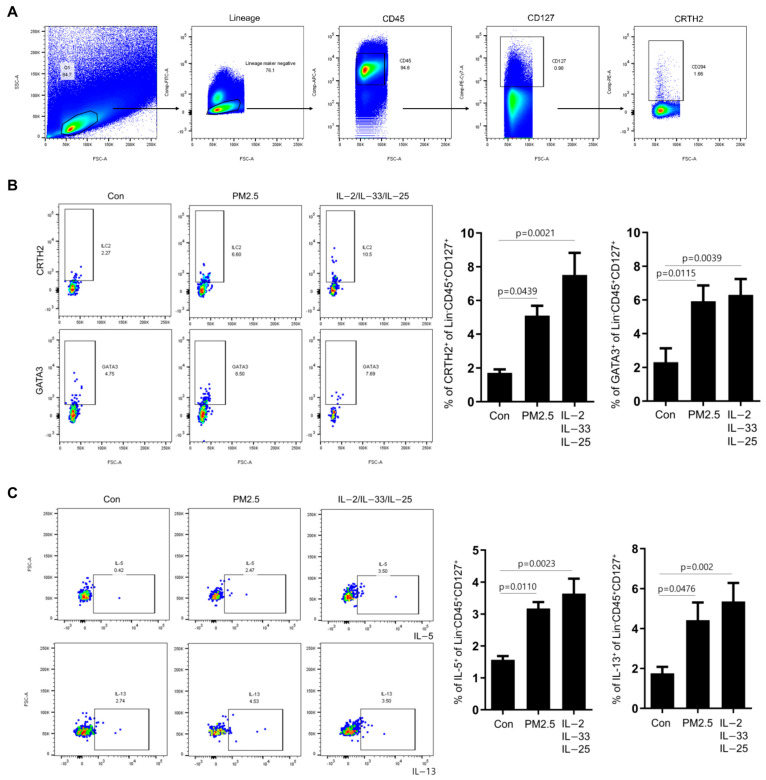
PM2.5 stimulates the induction of ILC2 in HNECs. (**A**) Gating strategies for flow cytometric analysis of ILC2 in the TMCs. (**B**) Lin cells were cultured with 5% plasma in PM2.5-CM, or as a positive control, in the presence of IL-2 (20 ng/mL), IL-33 (20 ng/mL), and IL-7 (20 ng/mL) for 3 days. The percentage of CRTH2 and GATA3 expression in Lin−CD45+CD127+ cells, and the ILC2 marker were determined using flow cytometry. (**C**) Intracellular IL-5+ Lin−CD45+CD127+ cell, IL-13+ Lin−CD127+ were measured using flow cytometry. The graphic data represent the mean ± standard error of mean (SEM; n = 5). Con = control. Statistical significance was determined using one-way ANOVA tests.

ILC2 can initiate the type 2 adaptive immune system and secretion of type 2 inflammatory cytokines in patients with CRSwNPs. Therefore, we investigated whether PM2.5-induced expression of type 2 cytokines upregulated ILC2. Accordingly, we characterized the phenotypes of ILC2 in TMCs. TMCs were gated in Lin (CD3, CD11c, CD14, CD16, CD19, CD20, CD56, and FceR1a)-negative and CD45+CD45+ cells, and CRTH+CD129+ cells were identified as the ILC2 population (Figure 4A). Lin-cells were isolated and stimulated with PM2.5-CM or IL-2, IL-33, IL-25 to induce ILC2, and the phenotypes of ILC2 were examined. PM2.5-CM stimulation significantly increased the ILC2 proportion and the expression of GATA3, a transcription marker of ILC2 (Figure 4B). Similarly, PM2.5-CM stimulation significantly increased IL-13 (*p* = 0.002) and IL-5 (*p* = 0.0023) expression in CD129+ cells (Figure 4C). GATA3 expression in Lin-CD45+CD127+CD294+ cells were examined following PM2.5-CM or IL-2, IL-33, and IL-25 stimulation to confirm whether the cells gated as Lin-CD45+CD127+CD294+ belonged to the ILC2 population. GATA3 was highly expressed in Lin-CD45+CD127+CD294+ cells in the control, PM2.5-CM, and positive control groups (Supplementary Figure S1). Additionally, nasal tissues were collected from patients with CRSwNP and the expression and proportion of Lin-CD45+CD127+CD294+ cells were examined to elucidate the effect of PM2.5 on tissue-resident ILC2, using flow cytometry (Supplementary Figure S2). Specifically, we examined IL-25/IL-33 expression and ILC2 phenotype in NP tissues cultured for 72 h with or without PM2.5. PM2.5 treatment increased the concentrations of IL-25 and IL-33 in the cell supernatants (Supplementary Figure S3A). Moreover, PM2.5 stimulations significantly increased ILC2 population and GATA3 expression in nasal tissues (Supplementary Figure S3B). Overall, these results suggest that PM2.5 stimulations can activate type 2 immune response and induce ILC2 population in the nasal mucosa of patients with CRSwNP.

### 2.5. Effect of RA on PM2.5-Induced Immune Response by ILC2

**Figure 5:**
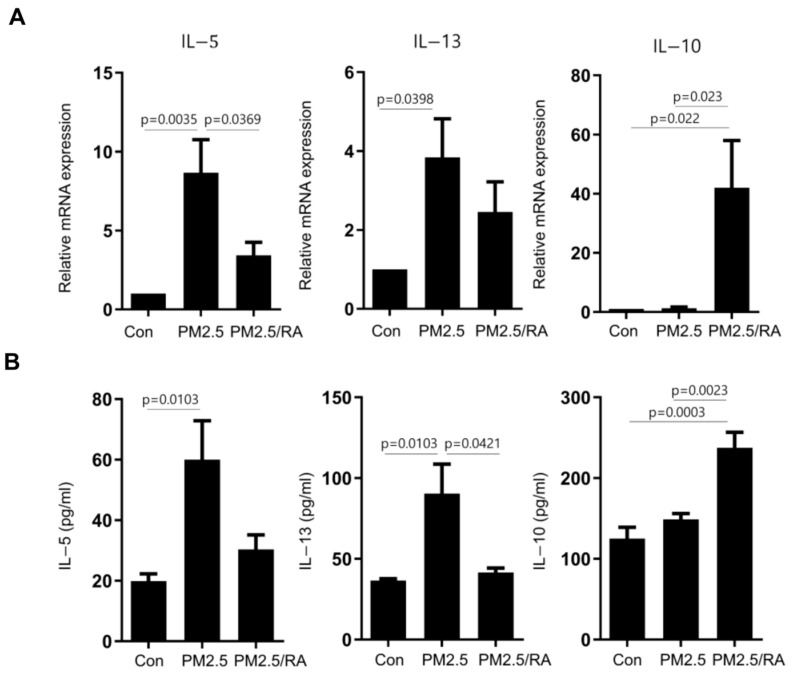

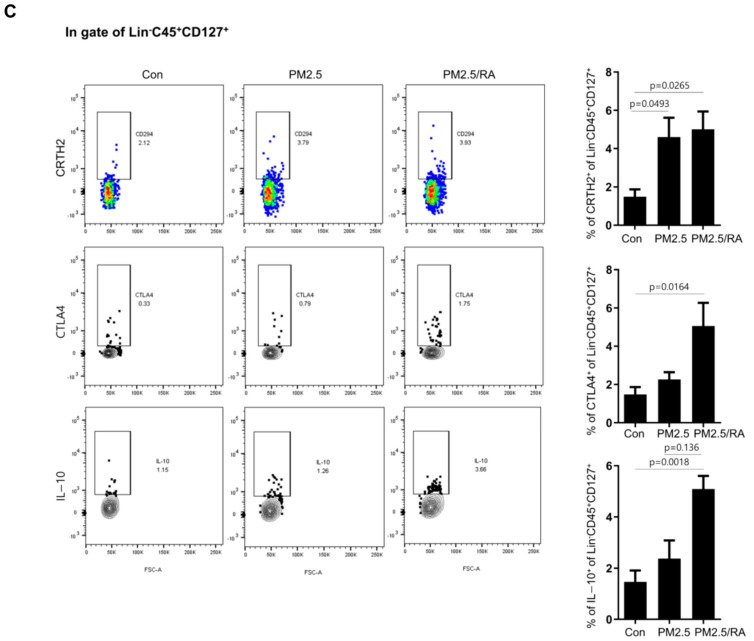
Effect of retinoid acid (RA) on PM2.5-induced ILC2 expression. (**A**) The mRNA expression of IL-4, IL-5, and IL-13 in Lin− cells was analyzed using qRT-PCR. (**B**) IL-4, IL-5, and IL-13 levels in the supernatant were analyzed using CBA assay. (**C**) CRTH2+CTLA4+ and intracellular IL-10+ expression in Lin−CD45+CD127+ cells was analyzed using flow cytometry (n = 6). Data are shown as means ± standard error of mean (SEM). Con = control. Statistical significance was determined using one-way ANOVA tests.

ILC2s are highly plastic cells that respond to changes in their microenvironments. RA treatment promotes the IL-10-producing phenotype of ILC2. Therefore, we investigated whether RA treatment could modulate PM2.5-CM-induced expression of ILC2. RA treatment significantly suppressed PM2.5-CM-induced upregulation of IL-5 and IL-13 expression in Lin cells at both the mRNA and protein levels. In contrast, RA treatment significantly upregulated IL-10 expression at both the mRNA and protein levels (Figure 5A,B). Although the ILC2 population was unaffected, RA treatment increased the expression of CTLA4, which is a phenotype of ILCreg (*p* = 0.0164), and the expression of IL-10 in Lin−CD45+CD127+ cells (*p* = 0.0018) (Figure 5C). Overall, these findings suggest that RA attenuates type 2 immune response via modulation of the ILC2 phenotype.

## 3. Discussion

CRSwNP is primarily characterized by type 2 inflammation, a condition influenced by both allergic and non-allergic factors. This inflammatory response is a result of the combined efforts of the immune system’s innate and adaptive components, especially those involving group 2 ILC and Th2 lymphocytes. These cells are key players in initiating, sustaining, and escalating inflammation through their production of type 2 cytokines, such as IL-4, IL-13, and IL-5 [24,25,26,27]. Moreover, ILC2 and Th2 cells, alongside mast cells, basophils, and eosinophils, are significant sources of other inflammatory agents, including IL-4, IL-13, IL-5, prostaglandin D2, and cysteinyl leukotrienes, which further contribute to the inflammatory milieu in CRSwNP.

ILCs are highly plastic cells that can transform to different phenotypes in response to environmental signals. Specifically, ILC2-mediated inflammation is characterized by increased production of type 2 cytokines, such as IL-4, IL-5, and IL-13 [28]. ILC2s also express cytokine receptors on their surfaces, among which IL-25 and IL-33 are major type 2 cytokine inducers in ILC2s [28]. ILC2s play an important role in type 2 inflammation in CRSwNP, and their activation may be restricted to the nasal mucosa of patients with CRSwNP [29,30]. Additionally, PM can stimulate lung ILC2s to produce high levels of IL-5 and IL-13 [31]. Meanwhile, recent studies demonstrated that RA treatment may suppress type 2 immune responses by increasing the population of ILCregs, and that it also increased fibrinolysis in NP tissues [22,23]. In the present study, we showed that PM2.5 exposures in NP tissues could increase type 2 immune response and upregulate the expression of epithelial-cell-derived cytokines such as IL-33 and IL-25. Moreover, an increase in the ILC2 population in NP tissues following PM2.5 exposures was observed, and it was determined that RA treatment could suppress the PM2.5-induced type 2 immune response by increasing the population of ILCregs. Collectively, these findings suggest that PM2.5 exposures may enhance type 2 immune response via an increased ILC2 population in NP tissues. Additionally, we found that RA treatment could ameliorate fine-dust-induced exacerbation of CRSwNP by modulating the innate immune response.

Generally, it is well-known that PM influences the development and exacerbation of lower respiratory tract disease. Prior studies demonstrated that short-term PM exposure is strongly associated with lower respiratory tract disease, such as chronic obstructive pulmonary disease, asthma, and pneumonia [32,33]. Meanwhile, one recent study also reported a close relationship between PM2.5 exposure and CRS development [34]. In the upper airway, PM can induce epithelial-to-mesenchymal transition (EMT), which involves the loss of epithelial polarity and junctional proteins [35]. Based on these facts, we thought that PM-induced EMT may influence the development of CRSwNP, the most severe type of CRS. Thus, firstly, we wanted to examine whether or not PM could aggravate the immune response of CRSwNP. Our animal study showed that PM2.5 aggravated the pathologic damage in the NP mouse model via enhanced type 2 inflammation.

CRSwNP is characterized by the influence of cytokines IL-5 and IL-13, originating from Th2 cells, ILC2 cells, and mast cells. Type 2 cytokines stimulate inflammatory cells involved in the pathogenic mechanism, including mast cells, basophils, and eosinophils [36]. Thus, the compelling significance of IL-5 and IL-13 in NP formation is well-established, regardless of whether type 2 cytokines originate from ILC2, Th2 cells, or mast cells and basophils. It is also widely acknowledged that the nasal microbiome experiences perturbations in the context of CRS. Some microorganisms, such as fungal species and Staphylococcus aureus, have been identified as potential contributors to inflammation in certain patient cohorts [36]. Specifically, Staphylococcus aureus is linked to the development and persistence of CRS, notably influencing the occurrence of nasal polyps (CRSwNP). The extensive range of exotoxins produced by S. aureus plays a crucial role in enabling the bacterium to maintain its infection and spread across various regions of the human body. Another study indicated that neutralizing antibodies specifically targeting Leukocidin ED could offer a significant supplementary approach for combating S. aureus infections [37].

Additionally, NP tissues were one of the first tissues in which ILC2s were discovered, and several studies have reported an increased ILC2 population in NP tissues [25,37,38,39,40]. Therefore, epithelial cell damage caused by infectious agents or allergens could result in the release of IL-25, IL-33, and thymic stromal lymphopoietin, which activates ILC2s [41,42,43]. Accordingly, we hypothesized that fine-dust-induced damage to epithelial cells in NP tissues may increase the ILC2 population and result in exacerbating type 2 immune response in patients with CRSwNP. In the present study, we investigated the effects of PM2.5 exposures on HNECs obtained from NP tissues. Consistent with previous findings [30,39,40], we found that PM2.5 exposures could increase the ILC2 population and upregulate the type 2 inflammation in NP tissue.

RA, a vitamin A metabolite, plays a key role in maintaining immune homeostasis during inflammatory responses by contributing to antibody synthesis [44]. A previous study described that retinoic acid receptor alpha (RARα), which is the high-affinity receptor for RA, could modulate Th2 cells through multiple mechanisms, including Th2 cell intrinsic augmentation of proliferation and IL-5 expression [45]. In addition, RA is a potent inducer of epithelial tissue plasminogen activators, particularly in patients with aspirin-exacerbated respiratory diseases [23]. It also induces homing of T, B, and innate immune cells, such as ILCs [43], and modulates ILC function. Combined RA with IL-2 treatment contributes to IL-5 and IL-13 upregulation in ILC2, and IFN-γ upregulation in ILCs 1 and 3, which are important for the functional maintenance of ILCs in allergic and inflammatory diseases [46]. Additionally, RA induces IL-10-producing ILCregs, which downregulate type 2 immune response and suppress ILC2 population. Similar to these previous findings, in this study, we observed that RA treatment could promote the expression of ILCregs, the anti-inflammatory phenotype of ILC. Interestingly, RA treatment significantly increased the proportion of ILCregs in Lin-CD45+CD127+ cells. Moreover, RA treatment downregulated IL-5 and IL-13 expression and upregulated IL-10 expression in ILC2-related inflammatory conditions caused by PM2.5 exposures. However, we have identified some critical limitations in this study. Initially, the absence of ILC2 samples from the NP tissues of patients limits our ability to conclusively determine the therapeutic efficacy of RA on inflammation exacerbated by PM2.5 in CRSwNP. Therefore, subsequent research should focus on exploring the therapeutic actions of RA against ILC2-driven inflammation induced by PM2.5, incorporating ILC2 samples from human NP tissues for a more in-depth analysis. Moreover, this study does not explore the mechanisms through which PM2.5 enhances the expression of type 2 cytokines, IL-33, and IL-25, leaving the precise pathway by which PM2.5 augments Th2-driven inflammation in CRSwNP unelucidated.

## 4. Materials and Methods

### 4.1. Mouse Model of Nasal Polyp and PM2.5 and PM10 Exposure

All animal experiments were reviewed and approved by the Institutional Review Board of Hallym Medical University Chuncheon Sacred Hospital (Chuncheon, Republic of Korea; IRB No.: 2020-50) and were performed under strict governmental and international guidelines for animal experiments. NPs were induced in mice according to previously described procedures [35,36]. Two types of fine dust were used in this study. For PM2.5, diesel PM Standard Reference Material (SRM) 1650b manufactured by the National Institute of Standards and Technology (NIST, Gaithersburg, MD, USA) was used, and for PM10, ERM-certified FDP reference material ERM-CZ100 (PM10-like) was used. They were purchased from SIGMA-ALDRICH (St. Louis, MO, USA). Briefly, 4-week-old wild-type (WT, BALB/c, Female) mice were divided into PBS-treated (control, n = 6), nasal polyp model (POYLP, n = 9), PM2.5-exposed NP model (POLYP + PM 2.5, n = 14), and PM10-exposed NP model (POLYP+PM 10, n = 14) groups. Mice with NP were immunized with an injection of 25 µg of ovalbumin (OVA; Sigma-Aldrich, St. Louis, MO, USA) dissolved in 300 µL complete adjuvant on days 0 and 5 (Figure 1A). At 1 week after the last injection, mice were challenged via instillation with 3% OVA in 40 µL of PBS for 5 consecutive days. Subsequently, prolonged nasal stimulation was maintained via instillation with 3% OVA in 40 µL for 12 weeks, and 20 ng of SEB in 20 µL of PBS was intranasally administrated once a week for the last 8 weeks. To examine the effects of PM2.5 and PM10, 25 µg of PM2.5 and PM10 in 20 µL of PBS were intranasally administrated twice a week for the last 8 weeks.

### 4.2. Mouse Nasal Tissue and Lavage Fluid Preparation

Mouse nasal mucosal tissue was collected, placed in tubes containing ceramic beads, and homogenized in easy-BLUE reagent (Intron Biotechnology, Seongnam, Republic of Korea) using Bead Ruptor Elite (Omni International, Kennesaw, GA, USA). Total RNA was extracted according to the manufacturer’s instructions. To isolate nasal lavage fluid, the nasal cavities were lavaged with 200 µL of PBS twice and the nasal lavage fluid was harvested into a 1.5-mL Eppendorf tube. After centrifugation, the supernatants were stored at −80 °C for further analyses.

### 4.3. Tissue Processing for Histopathological Analysis

Mice were sacrificed 1 day after terminal instillation with fine dust, and their heads, including nasal cavity and sinuses, were removed, fixed in 4% paraformaldehyde, decalcified, and embedded in paraffin. The paraffin blocks were cut into 4-mm sections, which were stained with hematoxylin-eosin to evaluate polyp formation and epithelial disruption.

### 4.4. Nasal Tissue Preparation and Isolation and Culture of Human Nasal Epithelial Cells

NP tissue samples were obtained from patients with CRSwNP during endoscopic sinus surgery. This study was approved by the Institutional Review Board (IRB) of Hallym Medical University Chuncheon Sacred Hospital (IRB No. 2019-10-009), and all participants provided written informed consent. NP tissue was cut into small pieces and enzymatically digested with collagenase type I (Worthington Biochemical Corporation, Lakewood, NJ, USA) and DNaseI (Sigma-Aldrich, St. Louis, MO, USA) for 30 min at 37 °C under stirring, followed by incubation in high-glucose Dulbecco’s modified Eagle’s medium (Welgene Inc., Gyeongsan, Republic of Korea) supplemented with 100 U/mL of penicillin (Sigma-Aldrich) and 100 mg/mL of streptomycin (Sigma-Aldrich). After incubation, the epithelial layer was separated from the stroma using a cell strainer, and the dissociated epithelial layers were transferred into a 50-mL conical tube, centrifuged at 200× *g* for 5 min, and resuspended in Airway Epithelial Cell Growth Medium (AECGM, PromoCell, Heidelberg, Germany). Thereafter, the epithelial layer in AECGM was cultured in a humidified atmosphere (5% CO_2_) at 37 °C. At approximately 80% confluency, HNECs from the epithelial layer were detached with trypsin-EDTA 0.25% (Gibco, Waltham, MA, USA). NP tissues for PM2.5 stimulations were harvested and rinsed in an AECGM supplemented with 100 U/mL of penicillin and 100 mg/mL of streptomycin. The biopsies were cut into multiple pieces (0.05 g) and incubated in AECGM in the absence or presence of PM2.5 for 48 or 72 h.

### 4.5. PM2.5 Stimulations and Conditioned Medium Collection

Briefly, HNECs (3 × 10^5^) were seeded in 6-well plates and incubated overnight, followed by stimulation with PM2.5 (50 μg/mL) at 37 °C for 24 h. After 24 h, the conditioned media (CM) was collected and centrifuged at 13,000 rpm for 10 min, filtered using a 0.22 μm syringe filter, and stored at −80 °C until use. HNECs were exposed to PM2.5 (50 μg/mL) for 24 h, and the conditioned medium (PM2.5 CM) was centrifugated. The supernatants of cells cultured in PM2.5 CM or in the presence of interleukin-2 (IL-2), IL-7, and IL-33 were collected and centrifuged at 1500 rpm for 5 min at 4 °C. The levels of the cytokines IL-4, IL-5, and IL-13 in the CM were measured using a LEGENDPlex custom-made kit (BioLegend, San Diego, CA, USA), according to the manufacturer’s instructions. Measurements were performed using FACSCalibur (BD Biosciences, San Jose, CA, USA). Cytokine concentrations are expressed as pg/mL.

### 4.6. Cytometric Bead Array

The concentrations of interferon-γ (IFN-γ), tumor necrosis factor-α (TNF-α), IL-6, IL-4, IL-10, and IL-17A in the nasal lavage fluid of the mice were measured using a Cytometric Bead Array (CBA) Mouse Th1/Th2/Th17 Cytokine Kit (BD Biosciences). The supernatants of Lin cells cultured in PM2.5 CM or in the presence of IL-2, IL-7, and IL-33 were collected and centrifuged at 1500 rpm for 5 min at 4 °C. The concentrations of IL-4, IL-5, and IL-13 in the CM were measured using a custom-made LEGENDPlex kit (BioLegend), according to the manufacturer’s instructions. Measurements were performed using FACSCalibur flow cytometer (BD Biosciences). Cytokine concentrations are expressed as pg/mL.

### 4.7. Flow Cytometry

To exclude dead cells, the cultured Lin cells were stained with a viable fluorescent reactive dye (Invitrogen, Waltham, MA, USA). Additionally, the cells were stained with anti-human hematopoietic lineage antibody cocktail (eBioscience, San Diego, CA, USA), anti-CD45- allophycocyanin (APC, BioLegend), anti-CD294 (CRTH2)-phycoerythrin (PE, Invitrogen), anti-CD127-PE-cy7 (Biolegend), and anti-CTLA4-APC-Cy7 (Invitrogen). For intracellular cytokine staining, Lin cells were stimulated with 50 ng/mL of PMA (Sigma-Aldrich), 1 μg/mL of ionomycin (Sigma-Aldrich), and 1 μg/mL of brefeldin A (Invitrogen) for 4 h. Thereafter, cells were stained with antibodies against surface markers, fixed, permeabilized using intracellular (IC) Fixation Buffer (eBioscience), and stained with anti-IL-5-APC (BioLegend), anti-IL-13-PerCP/Cy 5.5 (BioLegend), and anti-IL-10-PerCP/Cy 5.5 (BioLegend). The cells stained were examined with a FACSCanto II instrument (Becton Dickinson, Los Angeles, CA, USA). For data analysis, BD FlowJo software (BD Bioscience, version 10.9.0) was utilized.

### 4.8. Lineage-Negative Cell Isolation and ILC2 Culture

It is known that ILC2s regulate the immune response and exist in lymphoid organs, including tonsils [47]. Thus, we performed the isolation of the human lineage-negative (Lin-) cells from TMC originating from tonsil tissue. For Lin- cell isolation, TMCs were depleted of T cells, B cells, NK cells, myeloid cells, granulocytes, and RBCs by labeling with biotin-conjugated anti-CD2, anti-CD3, anti-CD10, anti-CD11b, anti-CD14, anti-CD16, anti-CD19, anti-CD56, anti-CD123, and anti-CD235a antibodies using a Lineage Cell Depletion Kit (Miltenyi Biotec, Bergisch Gladbach, Germany). The concentrations of cytokines in the culture supernatants were determined using ELISA. The expression of CD45+ CD129+ CRTH2+ cells, IL-5+ ILC2s, and IL-13+ ILC2s among Lin− cells was determined using flow cytometry. To induce ILC2 in vitro culture, Lin cells (100,000 cells per well) were cultured in a medium containing 5% plasma in PM2.5-CM or in the presence of IL-2 (20 ng/mL; BioLegend) plus IL-7 (20 ng/mL; BioLegend) and IL-33 (20 ng/mL; BioLegend) in 96-well round-bottom plates for 3 days. In some experiments, all-trans RA (EMD Millipore) was added.

### 4.9. Enzyme-Linked Immunosorbent Assay (ELISA)

Cultured cells were lysed in RIPA buffer, and cell lysates were collected to measure protein production. The levels of IL-25, IL-33, and TSLP were measured using an ELISA kit (Solarbio Science & Technology, Beijing, China) and analyzed using a microplate reader (GloMax^®^ Discover Microplate Reader, Madison, WI, USA).

### 4.10. Quantitative Real-Time PCR (qRT-PCR)

The mRNA expression of cytokines in nasal tissue and HNECs was examined using qRT-PCR, as previously described [15]. Briefly, total RNA was extracted using an easy-BLUE reagent (Intron Biotechnology, Seongnam, Republic of Korea), followed by cDNA synthesis from 2 μg of total RNA using the AccuPower cDNA synthesis kit (Bioneer, Daejeon, Republic of Korea). The cDNA was amplified and quantified on a PCR system using SYBR Green Master Mix (Applied Biosystems, Foster City, CA, USA). The primer sequences used to amplify the specific genes are listed in Supplementary Table S1.

### 4.11. Data Analyses

In this study, we used the Shapiro–Wilk test to evaluate the normal distribution, due to small samples. After the normality test, we selected an independent *t*-test or Mann–Whitney U-test for between-group comparisons. Data for comparing three groups were also evaluated by one-way analysis of variance (ANOVA) followed by Tukey post-hoc tests. All experiments were performed in more than triplicate. This statistical approach facilitated the evaluation of variance in data sets, providing a robust methodology for comparative analysis in the research context. All statistical analyses were performed using GraphPad version 8.0.2. Statistical significance was set at *p* < 0.05.

## 5. Conclusions

In the present study, we found that PM2.5 plays an important role in eliciting inflammatory changes in patients with CRSwNP via enhanced type 2 immune response and increases ILC2 population. Additionally, our findings revealed that RA treatment could suppress PM2.5-induced type 2 immune response via increased ILCreg population. These findings suggest that RA might have a therapeutic effect on fine-dust-exacerbated ILC2-related type 2 inflammation in patients with CRSwNP.

## Data Availability

All data presented in this study are available in this article.

## Supplementary Materials

The following supporting information can be downloaded at: https://www.mdpi.com/article/10.3390/ijms25073856/s1.
