# Supplementary material for: Retinoic Acid Treatment Mitigates PM2.5-Induced Type 2 Inflammation: Insights into Modulation of Innate Immune Responses
Source: Int J Mol Sci. 2024 Mar 29;25(7):3856. doi: 10.3390/ijms25073856 (PMC11011870; doi:10.3390/ijms25073856)
Supplement: Supplementary file 1 [file ijms-25-03856-s001.zip › ijms-2853533-supplementary.pdf]

# Supplementary Materials

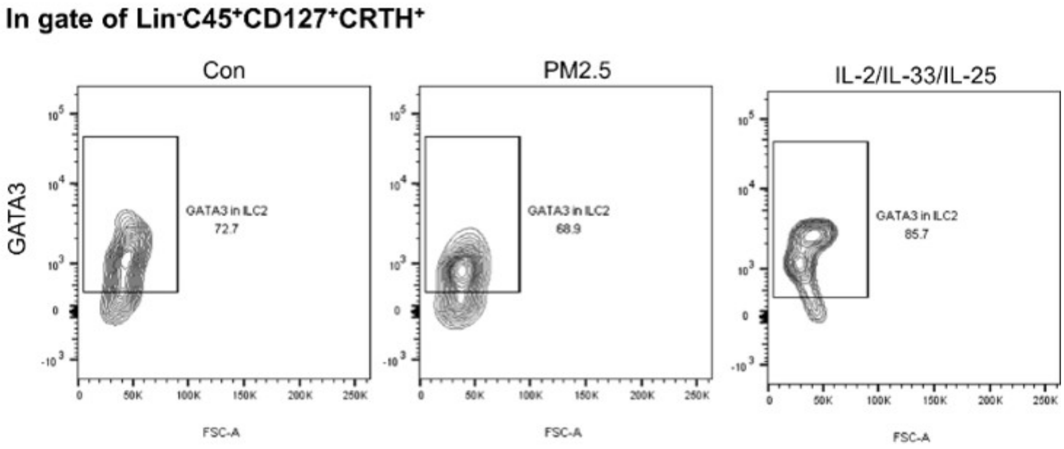


**Supplementary Figure 1.** Analysis of GATA 3 expression in CD45+Lin−CD127+CRTH2+ for ILC2s: Con = control.

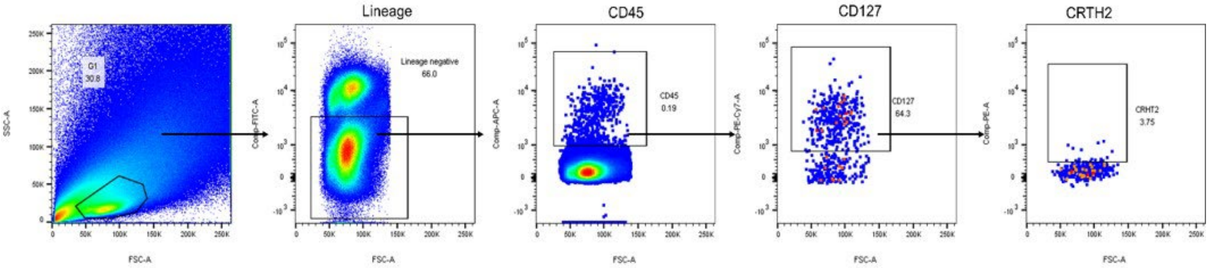


**Supplementary Figure 2.** Gating strategies of flow cytometric analysis of ILC2s in the nasal tissue

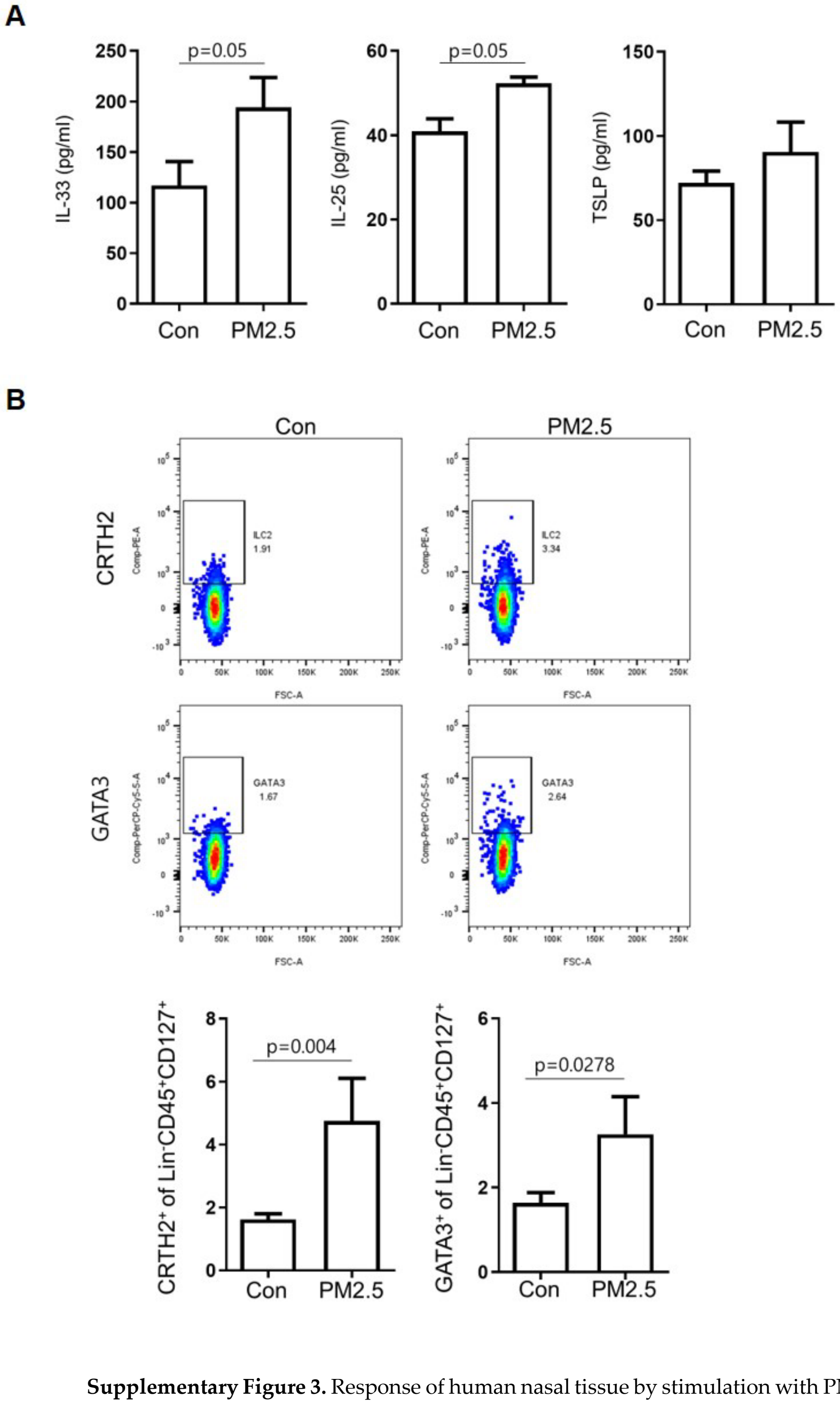


**Supplementary Figure 3.** Response of human nasal tissue by stimulation with PM2.5: Con = control. Statistical significance was determined using Mann–Whitney U test.

**Table S1.** Primers for real-time PCR.

| Genes | Forward 5′→3′ | Reverse 5′ →3′ |
|---|---|---|
| mouse GAPDH | CGTCCCGTAGACAAAATGGT | TTGATGGCAACAATCTCCAC |
| mouse TNF-$\alpha$ | GGCAGGTCTACTTTGGAGTCA | CACTGTCCCAGCCATCTTGTG |
| mouse IFN-g | AAAGAGATAATCTGGCTCTGC | GCTCTGAGACAATGAACGCT |
| mouse IL-4 | ACAGGAGAAGGGACGCCAT | GAAGCCCTACAGACGAGCTCA |
| mouse IL-6 | GAGGATACCACTCCCAACAGACC | AGTGCATCATCGTTGTTCATACA |
| mouse IL-10 | CACAAAGCAGCCTTGCAGAA | AGAGCAGGCAGCATAGCAGTG |
| mouse IL-17a | GCTCCAGAAGGCCCTCAGA | CTTTCCCTCCGCATTGACA |
| human β-actin | GGC CAT CTC TTG CTC GAA GT | GTG CTA TCC CTG TAC GCC TC |
| human IFN-g | TGCTCTCCTGTTGTGCTTCTCCAC | ATAGATGGTCAATGCGGCGTCC |
| human TNF-$\alpha$ | TGTAGCCCATGTTGTAGCAAACC | GAGGACCTGGGAG-TAGATGAGGTA |
| human IL-4 | CCGAGTTGACCGTAACAGACAT | GTCCTTCTCATGGTGGCTGTAG |
| human IL-5 | CAGGGAATAGGCACACTGGAG | GCACAGCCAGGACAAATATAGC |
| human IL-6 | AATTCGGTACATCCTCGACGG | GGTTGTTTTCTGCCAGTGCC |
| human IL-13 | CAAGGTCTCAGCTGGGGTAA | GGATATTCAGCCAGCTTCCCTT |
| human IL-25 | CCAGGTGGTTGCATTCTTGG | TGGCTGTAGGTGTGGGTTCC |
| human IL-33 | ACAGAATACTGAAAAATGAAGCC | CTTCTCCAGTGGTAGCATTTG |
| human TSLP | GGGCTGGTGTTAACTTAC-GACTTCA | TGAGTGGGACCAAAAGTAC-CGAGT |
